# Associations between novel genetic variants in the promoter region of *MALAT1* and risk of colorectal cancer

**DOI:** 10.18632/oncotarget.21507

**Published:** 2017-10-04

**Authors:** Yingjun Li, Chengzhen Bao, Simeng Gu, Ding Ye, Fangyuan Jing, Chunhong Fan, Mingjuan Jin, Kun Chen

**Affiliations:** ^1^ Department of Epidemiology and Health Statistics, Zhejiang University School of Public Health, Hangzhou, China; ^2^ Department of Public Health, Hangzhou Medical College, Hangzhou, China

**Keywords:** lncRNA, MALAT1, genetic variants, colorectal cancer

## Abstract

The metastasis-associated lung adenocarcinoma transcript 1 (*MALAT1*), a well-known long non-coding RNA, is involved in pathogenesis and progress of multiple tumors. However, no study has been performed to investigate the relationship between the genetic variants in promoter region of *MALAT1* and colorectal cancer risk. In this study, we conducted a two-stage case-control study to evaluate whether *MALAT1* genetic variants were associated with colorectal cancer risk. We identified that a single nucleotide polymorphism (SNP) rs1194338 was significantly associated with the decreased colorectal cancer risk with an odds ratio (OR) of 0.70 [95% confidence interval (CI) = 0.49-0.99] in the combined stage. The subsequently stratified analyses showed that the protective effect of rs1194338 was more pronounced in several subgroups. Furthermore, gene expression profiling analysis revealed overexpression of *MALAT1* mRNA in colorectal cancer tissue compared with normal controls. Confirmation studies with large sample size and further mechanistic investigations into the function of *MALAT1* and its genetic variants are warranted to advance our understanding of their roles in colorectal carcinogenesis, and to aid in the development of novel and targeted therapeutic strategies.

## INTRODUCTION

Long non-coding RNAs (lncRNAs), defined as RNA molecules greater than 200 nucleotides in length, have gained much attention due to their crucial roles as epigenetic regulators of gene expression [1]. Recently, increasing numbers of studies have reported that lncRNAs are dysregulated in many complex diseases, such as ischemic stroke [2], Alzheimer's disease [3], heart disease [4] and cancers [5, 6]. Especially, the metastasis-associated lung adenocarcinoma transcript 1 (*MALAT1*) which is transcribed from nuclear-enriched transcript 2 (NEAT2), is a novel 8.1kb long non-coding RNA [7]. It is located at chromosome 11q13.1, and is highly expressed in various types of human tissues. Progressing findings have suggested that *MALAT1* activates the development and progression of cancer by participating in multiple processes including cell proliferation, migration, invasion and apoptosis [7–10]. Also, Malakar *et al*. have indicated that *MALAT1* promotes hepatocellular carcinoma development by serine/arginine-rich splicing factor 1 (SRSF1) upregulation and mammalian target of rapamycin (mTOR) activation [11]. Lee *et al*. have validated that, when compared with normal cell lines and tissues, the expression of *MALAT1* is significantly elevated in various gastric cancer cell lines as well as gastric cancer tissues, and *MALAT1* is involved in gastric tumorigenesis by inhibiting cell apoptosis and promoting cell invasiveness via the epithelial-to-mesenchymal transition [12]. Further study has shown that *MALAT1* could promote gallbladder cancer development by acting as a molecular sponge to reduce the expression of miR-206 [13]. In colorectal cancer (CRC), the *MALAT1* level is highly expressed in tumor and is associated closely with CRC invasion and metastasis [14]; besides, *MALAT1* could motivate CRC cell proliferation, migration and invasion through PRKA kinase anchor protein 9 (AKAP-9) [15].

Recently, many epidemiologic studies have explored the link between genetic variants in lncRNAs and cancer susceptibility. Actually, it has been shown that among the identified genetic variants which are associated with multiple traits or diseases, 43% of them are intergenic and 45% are intronic according to a multifaceted analysis of published genome-wide association studies [16]. For example, the single nucleotide polymorphism (SNP) rs2147578 in *lnc-LAMC2-1:1* is significantly associated with increased risk for CRC occurrence by inhibiting miRNA binding [17]. Besides, novel SNP rs16941835 in lncRNA *RP11-58A18.1* has been newly identified to be associated with increased CRC risk in a genome-wide association study and meta-analysis [18]. Furthermore, Xue *et al*. have demonstrated that tag SNP rs7958904 in *HOTAIR* is related with decreased CRC risk and may act as a potential biomarker for predicting the risk of CRC [19]. Actually, several studies have demonstrated that lncRNA *MALAT1* polymorphisms are related with different disease susceptibility [20, 21]. Nevertheless, to the best of our knowledge, no study has been performed to evaluate the association between *MALAT1* genetic variants and the risk of CRC.

Otherwise, as with that SNPs in protein-coding genes could play important roles in the development of complex human diseases by affecting gene expression and function [22, 23]; polymorphisms in promoter region of functional lncRNAs might also be associated with disease susceptibility by affecting the stability and efficiency of transcription, resulting in modulation of their interaction partners [24, 25]. Therefore, a two-stage case-control study was conducted to investigate the association between SNPs in promoter region of *MALAT1* and CRC risk in a Chinese population.

## RESULTS

### Characteristics of study population

In the present study, we included a total of 821 CRC cases and 857 controls in two stages. There were no statistically significant differences between CRC cases and healthy controls in the combined stage relating to age, gender, body mass index (BMI), smoking and alcohol drinking (all *P*>0.05, Table 1). However, we found that higher percentage of patients reported a family history of cancer (*P*< 0.001) and non-tea drinking status (*P*=0.016) when compared with healthy controls. Besides, 48.84% of included cases were colon cancer and 51.16% were rectal cancer.

**Table 1:** The demographic and lifestyle-related characteristics between the colorectal cancer cases and controls

	Stage 1		Stage 2		Combined stage ^a^		*P*
	Case (n = 320)	Control (n = 319)	Case (n = 501)	Control (n = 538)	Case (n = 821)	Control (n = 857)	
Age (mean ± SD)	65.76±10.23	65.30±9.76	62.73±10.99	62.26±10.53	63.91±10.79	63.39±10.35	0.316
Gender, *N* (%)							
Male	168 (52.50)	157 (49.22)	262 (52.30)	285 (52.97)	430 (52.38)	442 (51.58)	0.743
Female	152 (47.50)	162 (50.78)	239 (47.70)	253 (47.03)	391 (47.62)	415 (48.42)	
BMI, *N* (%)							
<18.5	27 (8.44)	17 (5.33)	47 (9.38)	46 (8.55)	74 (9.01)	63 (7.35)	0.239
18.5∼23.9	216 (67.50)	201 (63.01)	339 (67.66)	363 (67.47)	555 (67.72)	564 (65.93)	
24∼27.9	61 (19.06)	85 (26.65)	99 (19.76)	113 (21.00)	160 (19.37)	198 (23.10)	
≥28	16 (5.00)	16 (5.02)	16 (3.19)	16 (2.97)	32 (3.90)	32 (3.62)	
Family history of cancer, *N* (%)							
No	257 (80.31)	287 (89.97)	394 (78.64)	450 (83.64)	651 (79.29)	737 (86.00)	**<0.001**
Yes	63 (19.69)	30 (9.40)	107 (21.36)	88 (16.36)	170 (20.71)	118 (13.77)	
Smoking, *N* (%)							
No	208 (65.00)	220 (68.97)	316 (63.07)	330 (61.34)	524 (63.82)	550 (64.18)	0.882
Yes	112 (35.00)	99 (31.03)	183 (36.53)	206 (38.29)	295 (35.93)	305 (35.59)	
Alcohol drinking, *N* (%)							
No	247 (77.19)	248 (77.74)	359 (71.66)	386 (71.75)	606 (73.81)	634 (73.98)	0.783
Yes	72 (22.50)	68 (21.32)	138 (27.54)	145 (26.95)	210 (25.58)	213 (24.85)	
Tea drinking, *N* (%)							
No	200 (62.50)	193 (60.50)	294 (58.68)	273 (50.74)	494 (60.17)	466 (54.38)	**0.016**
Yes	120 (37.50)	126 (39.50)	201 (40.12)	259 (48.14)	321 (39.10)	385 (44.92)	
Tumor site							
Colon	157 (49.06)		244 (48.70)		401 (48.84)		
Rectal	163 (50.94)		257 (51.30)		420 (51.16)		

SD, standard deviation; BMI, body mass index; N, number

^a^ Combine Stage 1 and Stage 2 as the combined stage

### Associations of SNPs and CRC risk

Table 2 shows the effects of four SNPs (i.e. rs4102217, rs591291, rs1194338 and rs600231) polymorphisms on CRC in Stage 1. The genotypes distributions of four SNPs among the controls were in accordance with Hardy-Weinberg equilibrium (HWE) (*P*>0.01). Subsequently, we applied three genetic effect models to evaluate the associations between the selected SNPs and the risk of CRC. However, no statistically significant association between the four SNPs and CRC risk was observed. After adjustment for age, gender, BMI, smoking, alcohol and tea drinking, we found that genetic effect of SNP rs1194338 in dominant model was just barely insignificantly associated with decreased CRC risk (*P*=0.113; OR = 0.77, 95% CI = 0.55-1.06). Besides, rs1194338 CA genotype, but not AA genotype, had a near-borderline significant association with decreased risk of CRC, compared with CC genotype (*P*=0.101; OR = 0.75, 95% CI = 0.54-1.06).

**Table 2:** Association of the selected SNPs with colorectal cancer risk in Stage 1

Variable	Case (%) (n=320)	Control (%) (n=319)	Model 1 ^a^	Model 2 ^b^	*P*-value ^c^
			OR (95% CI)	OR (95% CI)	
rs4102217 (*P*_HWE_= 0.20)					
Codominant model					
GG	235 (73.67)	246 (77.12)	1.00	1.00	
GC	79 (24.76)	71 (22.26)	1.16 (0.81-1.68)	1.14 (0.78-1.66)	0.506
CC	5 (1.57)	2 (0.63)	-	-	-
Dominant model					
GG	235 (73.67)	246 (77.12)	1.00	1.00	
GC+CC	84 (26.33)	73 (22.88)	1.20 (0.84-1.73)	1.19 (0.82-1.73)	0.366
Recessive model					
GG+GC	314 (98.43)	317 (99.37)	1.00	1.00	
CC	5 (1.57)	2 (0.63)	-	-	-
rs591291 (*P*_HWE_= 0.63)					
Codominant model					
CC	119 (37.42)	112 (35.44)	1.00	1.00	
CT	150 (47.17)	156 (49.37)	0.90 (0.64-1.27)	0.86 (0.60-1.24)	0.421
TT	49 (15.41)	48 (15.19)	0.96 (0.60-1.54)	0.93 (0.57-1.52)	0.762
Dominant model					
CC	119 (37.42)	112 (35.44)	1.00	1.00	
CT+TT	199 (62.58)	204 (64.56)	0.92 (0.66-1.27	0.88 (0.63-1.23)	0.453
Recessive model					
CC+CT	269 (84.59)	268 (84.81)	1.00	1.00	
TT	49 (15.41)	48 (15.19)	1.02 (0.66-1.57)	1.01 (0.64-1.58)	0.977
rs1194338 (*P*_HWE_= 0.02)					
Codominant model					
CC	152 (47.50)	134 (42.54)	1.00	1.00	
CA	141 (44.06)	157 (49.84)	0.79 (0.57-1.10)	0.75 (0.54-1.06)	0.101
AA	27 (8.44)	24 (7.62)	0.99 (0.55-1.80)	0.86 (0.46-1.61)	0.639
Dominant model					
CC	152 (47.50)	134 (42.54)	1.00	1.00	
CA+AA	168 (52.50)	181 (57.46)	0.82 (0.60-1.12)	0.77 (0.55-1.06)	0.113
Recessive model					
CC+CA	293 (91.56)	291 (92.38)	1.00	1.00	
AA	27 (8.44)	24 (7.62)	1.11 (0.63-1.98)	1.00 (0.55-1.82)	0.992
rs600231 (*P*_HWE_= 0.44)					
Codominant model					
AA	118 (36.88)	111 (34.91)	1.00	1.00	
AG	152 (47.50)	160 (50.31)	0.89 (0.64-1.26)	0.83 (0.58-1.19)	0.320
GG	50 (15.62)	47 (14.78)	1.00 (0.62-1.61)	0.94 (0.57-1.54)	0.798
Dominant model					
AA	118 (36.88)	111 (34.91)	1.00	1.00	
AG+GG	202 (63.12)	207 (65.09)	0.92 (0.66-1.27)	0.86 (0.61-1.20)	0.376
Recessive model					
AA+AG	270 (84.38)	271 (85.22)	1.00	1.00	
GG	50 (15.62)	47 (14.78)	1.06 (0.69-1.64)	1.04 (0.66-1.63)	0.864

HWE, Hardy-Weinberg equilibrium

^a^ Crude Model

^b^ Adjusted for age, gender, BMI, smoking, alcohol and tea drinking

^c^ *P*-value for adjusted model

In addition, we identified that rs1194338 was significantly associated with CRC risk in Stage 2 (Table 3). After adjustment for potential confounders, rs1194338 variant allele A showed a statistically significant association with decreased CRC risk: the ORs were 0.63 (95% CI: 0.41-0.97) for codominant model and 0.62 (95% CI: 0.41-0.93) for recessive model. When combined these two stages, the AA genotype, but not CA genotype, had a statistically significant association with decreased risk of CRC, compared with CC genotype (*P*=0.045; OR = 0.70, 95% CI = 0.49-0.99).

**Table 3:** Association of rs1194338 with colorectal cancer risk in Stage 2 and combined stage

Variable	Case (%) (n=501)	Control (%) (n=538)	Model 1 ^a^	Model 2 ^b^	*P*-value
			OR (95% CI)	OR (95% CI)	
rs1194338 (*P*_HWE_= 0.67)					
Codominant model					
CC	237 (47.59)	247 (45.91)	1.00	1.00	
CA	216 (43.37)	220 (40.89)	1.02 (0.79-1.33)	1.06 (0.81-1.38)	0.690
AA	45 (9.04)	71 (13.20)	**0.66 (0.44-1.00)**	**0.63 (0.41-0.97)**	**0.036**
Dominant model					
CC	237 (47.59)	247 (45.91)	1.00	1.00	
CA+AA	261 (52.41)	291 (54.09)	0.94 (0.73-1.19)	0.95 (0.74-1.22)	0.685
Recessive model					
CC+CA	453 (90.96)	467 (86.80)	1.00	1.00	
AA	45 (9.04)	71 (13.20)	**0.65 (0.44-0.97)**	**0.62 (0.41-0.93)**	**0.021**
Combined stage					
	Case(%) (n=821)	Control (%) (n=857)			
rs1194338					
Codominant model					
CC	389 (47.56)	381 (44.67)	1.00	1.00	
CA	357 (43.64)	377 (44.20)	0.92 (0.75-1.13)	0.91 (0.65-1.27)	0.115
AA	72 (8.80)	95 (11.14)	0.75 (0.54-1.06)	**0.70 (0.49-0.99)**	**0.045**
Dominant model					
CC	389 (47.56)	381 (44.67)	1.00	1.00	
CA+AA	429 (52.44)	472 (55.33)	0.89 (0.74-1.08)	0.88 (0.72-1.07)	0.205
Recessive model					
CC+CA	746 (91.20)	758 (88.86)	1.00	1.00	
AA	72 (8.80)	95 (11.14)	0.82 (0.48-1.37)	0.75 (0.47-1.18)	0.213

HWE, Hardy-Weinberg equilibrium

^a^ Crude Model

^b^ Adjusted for age, gender, BMI, smoking, alcohol and tea drinking

^c^ *P*-value for adjusted model

**Figure 1:**
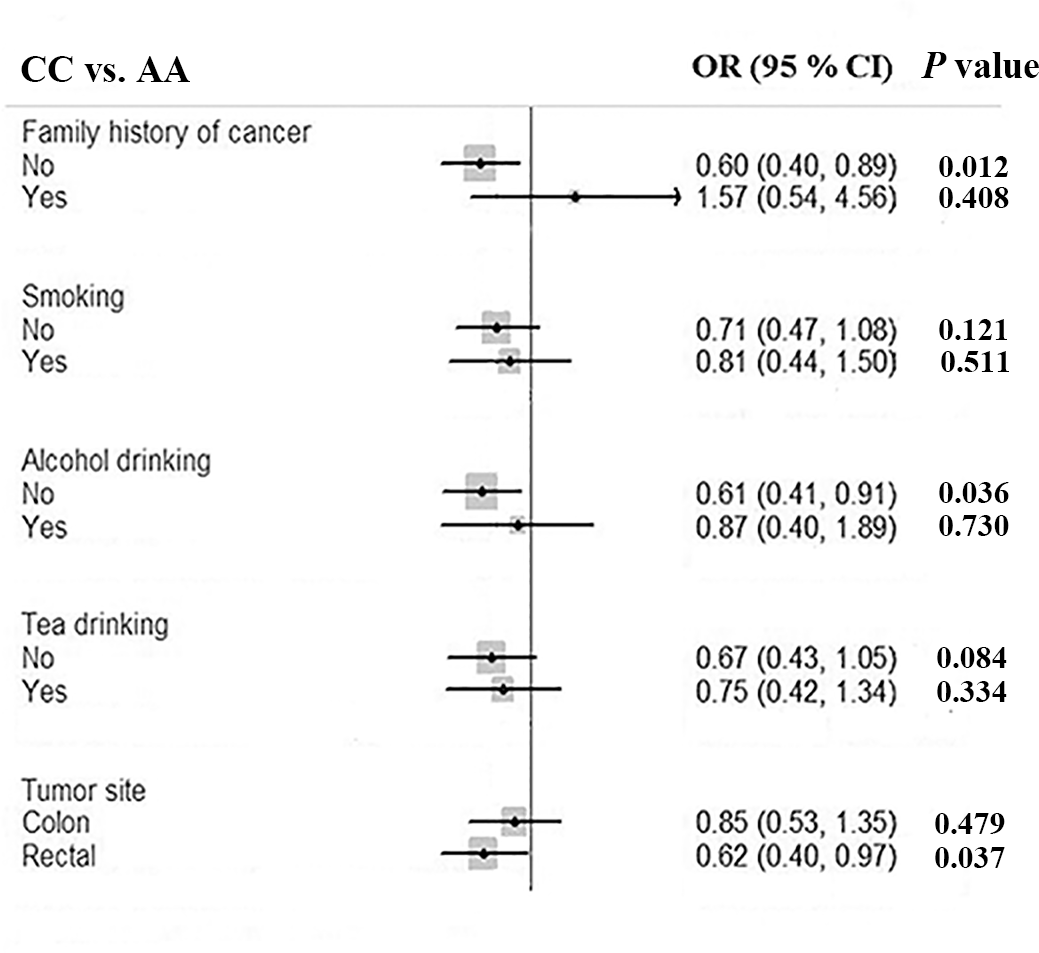
Forest plot shows odds ratio for the association between *MALAT1* rs1194338 polymorphism and the risk of colorectal cancer stratified by lifestyle-related characteristics and tumor site (AA vs. CC)

Furthermore, stratified analyses relating to lifestyle-related characteristics and tumor site were performed. More pronounced risk effect of homozygous variant genotype of rs1194338 were found in subgroup of no history of cancer (AA vs. CC: OR = 0.60, 95% CI = 0.40-0.89), no habit of alcohol drinking (AA vs. CC: OR = 0.61, 95% CI = 0.41-0.91), and rectal cancer (AA vs. CC: OR = 0.62, 95% CI = 0.40-0.97) (Figure 1).

### Functional relevance of rs1194338 on *MALAT1* expression level

**Figure 2:**
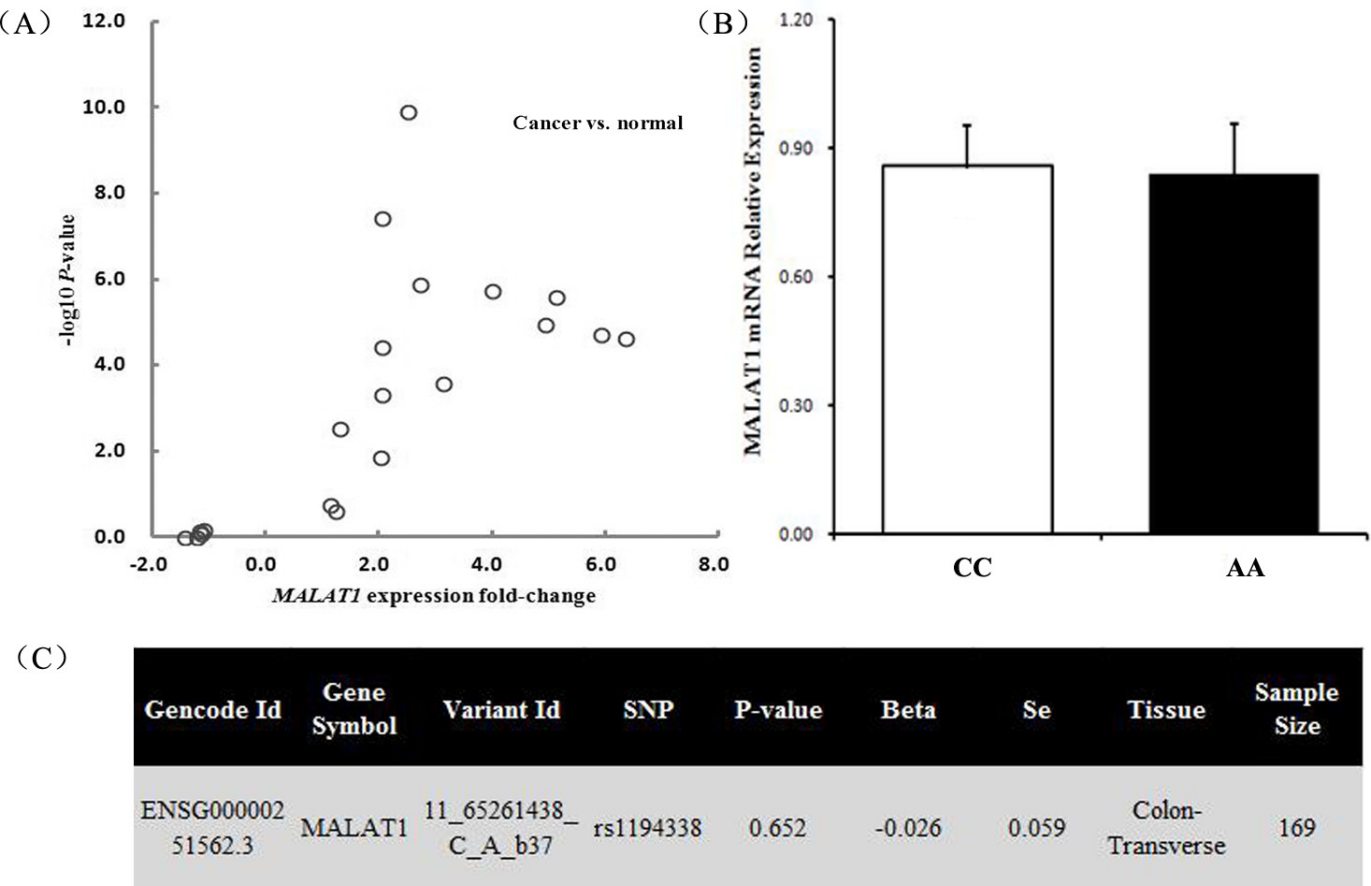
Functional relevance of rs1194338 on *MALAT1* expression level **(A)** Expression analysis of *MALAT1* in colorectal cancer tissue and normal controls from Oncomine database. Volcano plots were generated using the *P*-value and fold-change of each probe comparison; **(B)** relative expression level of *MALAT1* mRNA grouped by rs1194338 CC and rs1194338 AA genotypes in 71 colorectal cancer tissues: there was no statistically significant difference between CC and AA genotypes; **(C)** correlation between *MALAT1* mRNA expression and rs1194338 polymorphism in 169 transverse colon tissues according to the Genome-Tissue Expression (GTEx) database.

To further elucidate the potential molecular relevance of the association, the Oncomine expression profiling database was used to determine whether the expression of *MALAT1* mRNA was altered in CRC tissue relative to controls. The *P*-value and the fold-change of each probe comparison between CRC tissue and normal controls were extracted to generate a volcano plot. As shown in Figure 2A, *MALAT1* mRNA were overexpressed in CRC tissue compared with adjacent normal tissue or tissue from healthy controls. Moreover, the expression levels of *MALAT1* were evaluated in 71 CRC tissues samples. Among the 71 samples, 34 individuals carried the rs1194338 CC genotype, 29 carried the CA genotype, and 8 carried the AA genotype. There was no statistically significant difference of *MALAT1* expression between CC genotype and AA genotype in CRC tissues (Figure 2B). Thirdly, according to the Genome-Tissue Expression (GTEx) database [26], no statistically significant down-regulation of *MALAT1* mRNA expression with rs1194338 variant genotype (C/A+A/A) compared with the wild-type homozygous genotype (C/C, *P*=0.652, Figure 2C) was observed in 169 transverse colon tissues.

## DISCUSSION

Colorectal cancer is among the most common gastrointestinal malignant neoplasms and represents the third cause of cancer morbidity and the fourth cause of cancer mortality worldwide, with 1.4 million new cases and 0.7 million deaths estimated to have occurred in 2012 [27]. The pathogenesis of CRC is a complex process that is tightly controlled by multiple layers of regulatory mechanisms, which entail the accumulation of both genetic and epigenetic alterations in proliferating cells [28]. In the current study, a two-stage case-control study was performed to investigate the effect of four *MALAT1* SNPs on CRC risk. As a result, SNP rs1194338 in the promoter region of *MALAT1* was significantly associated with decreased CRC risk, especially in the subgroup of no history of cancer, no habit of alcohol drinking, and rectal cancer. Gene expression profiling analysis revealed increased expression of *MALAT1* in CRC tissue compared with normal controls. Taken together, this is the first study to explore the association between *MALAT1* genetic variants and CRC risk and provide further evidence for the involvement of *MALAT1* in CRC tumorigenesis.

Progressing findings have suggested a crucial role of lncRNAs in tumorigenesis. For example, *HOTAIR* could affect gene transcription and involve malignance by interacting with chromatin-remodeling complexes as well as recruiting these complexes to specific genomic DNA sequences [29, 30]. Besides, lncRNAs *PRNCR1* and *PCGEM1*, specifically interact with androgen receptor (AR) and strongly enhance androgen receptor-mediated gene activation in both ligand-dependent and ligand-independent manner [31]. Another lncRNA, *H19* could promote tumor metastasis by regulating critical events including the epithelial-to-mesenchymal transition and the mesenchymal-to-epithelial transition [32]. Some reports have shown that *MALAT1* could regulate the expression of metastasis-associated genes as well as cell cycle genes [33], and perform key roles in G1/S and mitotic progression [34]. Furthermore, *MALAT1*-depleted cells show cell cycle defects which are sensitive to the p53 levels, indicating that p53 is a main downstream mediator for the *MALAT1* activity [34].

Genetic variants in lncRNAs could be associated with the development and progression of multiple diseases by influencing their expressions or functions [35]. The promoter, a regulatory region of DNA located upstream of a gene, plays an important role in transcriptional regulation [36]. Thus, it is believed that genetic variants in promoter region can affect the expression, stability and subcellular localization of transcriptome, resulting in function changes and disease occurrence [37]. In our study, we observed the association between SNP rs1194338 in *MALAT1* promote region and the decreased risk of CRC. However, the rs1194338 polymorphism (C>A) did not affect the expression levels of *MALAT1* neither in transverse colon tissues nor in CRC tissues based on bioinformatics analysis and quantitative RT-PCR. We hypothesized that some of the common variants might change the cell-to-cell variability, temporal dynamics or cell cycle dependence of gene expression at the single-cell level, instead of influencing the average gene expression of a gene in a whole tissue [38]. It may also because of that many regulatory genetic variants display functionality only after pathophysiologically relevant immune stimuli. It is possible to more extensively resolve functional genetic variants and the specific modulated genes associated with cancer by considering the cellular and environmental context relevant to cancer [39]. Thus, we could not detect the direct correlation between the rs1194338 polymorphism and *MALAT1* mRNA expression level in the present study. Further studies are needed to investigate the precise mechanism underlying the function of the synonymous substitution.

There are some limitations in this study. One of the limitations is the recall bias, which is inevitable in case-control studies. However, as recall bias would not affect the genotype, it is of less concern in genetic association studies. On the other hand, the observed associations of our study might have insufficient statistical power due to the relatively moderate sample size [21]. However, we used a two-stage investigation to validate the association between the genetic variants and CRC susceptibility. Thirdly, information about clinical and pathological characteristics was unavailable in the current study, which might confine the representativeness of our findings. It is widely accepted that clinical and pathological characteristics are commonly related to many distinct molecular biological events, which could delineate the tumor development to some extent [40]. For example, the TNM staging could involve distinct molecular signatures reflecting the stepwise progression of CRC [41].

In conclusion, the findings from our two-stage population-based genetic association analysis provide the first evidence of the association between lncRNA *MALAT1* rs1194338 polymorphism and colorectal carcinogenesis. The variant genotype (AA) of rs1194338 decreased CRC risk. Furthermore, gene expression profiling analysis revealed overexpression of *MALAT1* mRNA in colorectal cancer tissue compared with normal controls. Confirmation studies with large sample size and further mechanistic investigations into the function of *MALAT1* and its genetic variants are warranted to advance our understanding of their roles in colorectal carcinogenesis, and to aid in the development of novel and targeted therapeutic strategies.

## MATERIALS AND METHODS

### Study subjects

This study was approved by the Medical Ethical Committee of Zhejiang University School of Medicine. All subjects enrolled were heritably unrelated ethnic Han Chinese. Details of the study population including recruitment details and participant characteristics have been described previously [42]. In brief, the registry information of this population was initially collected for a cohort study on CRC in 1989 in Jiashan County, Zhejiang Province, China. Meanwhile a cancer surveillance and registry system covering the whole county was established for reporting new cancer patients of CRC and all other kinds of cancers. Although there were no restrictions on patients’ age, gender or tumor stage, only those patients who were incident and histologically confirmed CRC, living in the study geographic area, mentally competent to complete the interview and with no previous diagnosis of familial adenomatous polyposis, ulcerative colitis or Crohn's disease were included in our study. Healthy controls with no previous history of cancer were recruited in parallel from the same population and were matched to cases by age (±5 years), gender and residential area. In total, 821 CRC cases and 857 healthy controls were recruited in two stages (320 cases and 319 controls for the Stage 1 which was recruited from 2012 to 2014, with additional 501 cases and 538 controls for the Stage 2 which was recruited from 2002 to 2010). Before participation, written informed consent was obtained from all the study subjects. Face-to-face interviews were conducted by trained interviewers, who administered a structured questionnaire relating to demographic characteristics and lifestyle-related factors. After interview, 5ml blood sample was collected into sodium citrate anticoagulant tubes and stored at -80°C for DNA isolation. Genomic DNA was isolated from peripheral blood samples for each study subject using the modified salting-out procedure [43].

CRC tissues from a total of 71 patients who had undergone curative surgery at Department of Gastrointestinal surgery, Hangzhou First People's Hospital were collected from July 2013 to December 2013. All the patients were pathologically confirmed as colorectal adenocarcinoma. The ttissues were immediately preserved in RNA Later® Stabilization Solution (Invitrogen, Carlsbad, CA, USA) after removal from the body and stored at -80°C.

### SNP selection and genotyping

We selected the tagSNPs which were located in 2000bp upstream region of *MALAT1* with minor allele frequencies (MAF) > 0.10 in Han Chinese Beijing from the 1000 Genome Projects. We also amplify the region to 5000bp as SNPs in this region could regulate gene expression by remote controlling the promoter region [44]. As a result, four tagSNPs (rs4102217, rs591291, rs1194338 and rs600231) were selected when linkage disequilibrium (LD) between pair-wise SNPs was with a minimum r^2^ of 0.80. Genotyping for all polymorphisms was performed by the MassARRAY molecular weight array analysis system (BioMiao Biological Technology Co., Beijing, China). Five percentage of samples were randomly selected for repeated detection and the concordance rate was > 99%.

### RNA isolation and quantitative RT-PCR

Total cellular RNA was isolated from each sample using a homogenizer (IKA®-Works Guangzhou, China) and TRIzol reagent (Invitrogen) and then purified using the RNeasy Mini Kit (Qiagen, Hilden, Germany) according to the manufacturer's protocol. A reverse transcription real-time PCR (RT-PCR) was conducted employing StepOnePlus instrument (Applied Biosystems, Foster City, CA, USA) to quantify relative *MALAT1* expression in these samples. The specific forward primer and reverse primer used for quantitative RT-PCR were 5′-TCCCTCAAGAGAACACAAGAAG-3′ and 5′-GGCGTATTTATAGACGGAGAAC-3′. The β-actin was selected as the endogenous control. The specific forward primer and reverse primer were 5′-GTGGCCGAGGACTTTGATTG-3′ and 5′-CCTGTAA CAACGCATCTCATATT-3′. We performed real-time PCR in StepOnePlus Real- Time PCR System (Applied Biosystems, Foster City, CA, USA) using SYBR Green Master Mix (Vazyme, Nanjing, China). All procedures were carried out in triplicate.

### Bioinformatics analysis

Furthermore, we applied several semi-automated bioinformatics tools to explore whether SNPs or their linked genetic variants are associated with a potential function that might affect the cancer development and progression. HaploReg [45] v4 and the GTEx database [26] from the ENCODE project [46] were used to identify the regulatory potential of candidate functional variants. The GTEx data were used to identify correlations between SNPs and transverse colon-specific gene expression levels.

### Expression analysis in colorectal cancer tissue

To determine the expression pattern of *MALAT1* in CRC, 20 probe comparisons in 10 studies, including Kaiser Colon [47], TCGA Colorectal (http://tcga-data.nci.nih.gov/tcga/), Zou Colon [48], Gaspar Colon [49], Graudens Colon [50], Hong Colorectal [51], Skrzypczak Colorectal [52], Skrzypczak Colorectal 2 [52], Sabates-Bellver Colon [53], Gaedcke Colorectal [54] from the publicly available cancer microarray database Oncomine (https://www.oncomine.org/; accessed on February 05, 2017) were used. The gene expression of *MALAT1* was compared between CRC tissues with normal tissues according to the standard procedures as previously described [55]. Volcano plots were generated using the *P*-value and fold-change of each probe comparison. Detailed information regarding the experimental protocols and tissue samples can be found in the Oncomine database.

### Statistical analysis

Differences in the distribution of selected demographic variables and genotypes of tagSNPs were evaluated by Pearson's chi-square test. Hardy-Weinberg equilibrium for each SNP among controls was tested using a goodness-of-fit chi-square test. The associations of each SNP and CRC susceptibility were estimated by using unconditional logistic regression analyses with odds ratios (ORs) and 95% confidence intervals (CIs). Multiple genetic models (codominant, dominant and recessive models) were applied to assess the significance of SNPs. Those tagSNPs with a *P* value less than 0.20 in Stage 1 were further studied in Stage 2. Pooled-analyses were conducted to estimate the combined effect of the two stages. The relative expression levels of *MALAT1* in 71 CRC tissues were calculated by the following formula: (levels of *MALAT1*-levels of β-actin)/ levels of β-actin. The one-way analysis of variance was applied to test statistically difference among the expression of *MALAT1* with diverse rs1194338 genotypes. A *P*-value of less than 0.05 for two-side was considered statistically significant. All analyses were conducted with SAS 9.2 software (SAS Institute, Cary, NC, USA) and Stata 11.2 (STATA Corp, College Station, Texas).
